# An integrated review: connecting Covid-era hospital visiting policies to family engagement

**DOI:** 10.3389/fpubh.2023.1249013

**Published:** 2023-09-01

**Authors:** Jennifer D. Morgan, Priscilla Gazarian, Laura L. Hayman

**Affiliations:** ^1^Manning College of Nursing and Health Science, University of Massachusetts Boston, Boston, MA, United States; ^2^Massachusetts General Hospital, Boston, MA, United States; ^3^Brigham and Women's Hospital, Boston, MA, United States

**Keywords:** family engagement, informal caregiver, integrative review, patient-family centered care, hospital visitor policy

## Abstract

**Introduction:**

Family engagement and patient-family-centered care are vitally important to improve outcomes for patients, families, providers, hospitals, and communities. Both constructs prioritize providers forming partnerships with patients and their families. The domains of family-engaged care include presence, communication, shared-decision making, family needs, contribution to care, and collaboration at the institutional level. This integrative review describes the extent to which the domains of family engagement are present in the literature about Covid-era hospital visiting policies.

**Methods:**

A search of four databases resulted in 127 articles and one added through data mining. After review, 28 articles were synthesized and analyzed into an integrative review of family engagement in the hospital with Covid-era visiting policies as the backdrop.

**Results:**

The 28-article review resulted in an international, multidisciplinary perspective of diverse study designs. The review’s sample population includes 6,984 patients, 1,126 family members, 1,174 providers, 96 hospitals, 50 health centers, 1 unit, and 257 documents. While all the domains are represented, presence is the prevailing domain, identified in 25 out of the 28 (89%).

**Discussion:**

Presence is recognized as facilitating the other domains. Because the concept of collaboration is largely absent in the literature, it may provide healthcare institutions with a growth opportunity to facilitate and promote family engagement. This review is the first step in operationalizing family engagement in the hospital setting, especially when presence is challenging.

## 1. Introduction

Family engagement has been identified as a construct that is vitally important to successful outcomes for patients, families, providers, hospitals, and communities. Hospitals often are the setting of new or worsening diagnoses for older and seriously ill patients. Family, as defined by the patient, refers to a trusted individual or group of individuals and does not necessarily reflect a legal relationship. Family engagement improves healthcare quality and safety and, therefore, patient outcomes in the hospital (1, 2). Family engagement at the end of a patient’s life has a broader scope of impact than on the patient alone. It is an antecedent and an attribute of a good death, facilitating healthy bereavement for families and job satisfaction for providers (3). In addition to improving health outcomes for the patient, family engagement also benefits the hospital. Families provide information, context, care coordination, and help with the transition home (4). Family engagement decreases readmission rates (5) and reduces overall healthcare utilization (6).

The constructs of Family Engagement and Patient and Family Centered Care overlap and run along a continuum that includes passive and active family involvement (7). With older adults, family engagement can be episodic, as seen in a new cancer diagnosis, or progressive, as seen in a dementia diagnosis (8). The six foundational components of the family engagement construct are presence, meeting family needs, communication, shared decision-making, contribution to care, and collaboration (7, 9). While the components of family engagement may require different levels of family involvement, they are not linear or discreet and are, in fact, interdependent (7). The physical presence of the family bedside, the least active of the domains, is often the precursor to the other components. The second component, meeting family needs or respect, focuses on building an effective partnership between families and providers by recognizing the family’s need for information, comfort, or resources (7, 9). Communication or information sharing between family and provider is a third family engagement component (7, 9). Shared decision-making is the fourth component of family involvement and is considered a more active form of family engagement that cannot be wholly unbraided from communication (7). The fifth, contribution to care, is the most active family involvement and encompasses physical and emotional support that the family may give the patient (7). The final component is collaboration and includes the family as a stakeholder at the institutional and policy level (9).

Family engagement is an invaluable resource to the community worldwide. For example, in 2020 in the United States, over 53 million adults reported being informal caregivers, an increase from 43.5 million caregivers in 2015 (10). In Massachusetts alone, informal caregivers provide almost 800 million hours of care, translating to nearly $12 billion annually (11). In the United States and other parts of the world, caregiving is not shared equally across the population. Most caregivers are women (12), and in the United States, black caregivers provide approximately 400 more hours a year than white caregivers (13). In addition to the gender and racial inequities that may exist, there are geographic disparities. Caregivers in rural areas have less access to support, making the intensity and burden greater in those areas (14). How informal caregiving translates to family engagement in the hospital is unknown.

Until the pandemic, restrictive visiting policies were often only seen in critical care units, where patient care is particularly complicated. However, in January 2020, the World Health Organization (WHO) declared SARS-CoV-2, often called Covid, an international public health emergency and went on to give operational guidance to hospitals to contain the spread of the virus by severely restricting both the number of visitors and the length of the visiting period (15). Visitor policies vary from one region to another, from one institution to another, and even within a particular institution between units. The extent to which the Covid-era visiting policies affect family engagement in the hospital is unknown. The dramatic change to visiting policies may offer an opportunity to learn more about family engagement in the hospital setting, revealing which domains are underutilized and, therefore, may offer the best growth opportunities. The integrative review aims to describe the extent to which the domains of family engagement are present in the literature about visiting policy; what domains the literature focus on, and where might the gaps in the literature be.

## 2. Framework

Integrative reviews synthesize empirical and theoretical literature to build nursing science and health policy (16). This review is guided by the Conceptual Model of Nursing and Health Policy (CMNHP). It focuses on the unique role of nursing within the multidisciplinary health policy domain (17). CMNHP recognizes nurses’ critical role in creating and implementing health policy (17). This review focuses on hospital visiting policies at the first level, which centers on individuals, families, and communities (17).

## 3. Methods

One researcher (JM), a clinical nurse and doctoral student, searched four databases, CINAHL, PubMed, Medline EBSCO, and Ovid. After meeting with a healthcare librarian, the search terms “hospital visitor policy” and “hospital visiting policy” were used to keep the results broad. To keep the search unbiased, the researcher avoided using the terms “restrictive” and “no-visiting.” The range of dates is narrow, including literature from 2020 to early 2023, to ensure that the results capture the Covid-era visiting policies. Articles were included if published in English and covered the adult population. Articles that were pre-Covid or were set outside of a hospital setting were excluded. Articles related to the pediatric population were also excluded because there are different ethical and practice considerations with pediatric populations related to family engagement and visiting policies. The researcher met regularly from conception through completion with the two other authors (LH and PG), experienced researchers, professors, and experts in health policy and serious illness. Questions and concerns were settled by consensus. Additionally, the research findings were presented to a group of peers to identify questions, concerns, and oversites.

## 4. Search results

**Figure 1:**
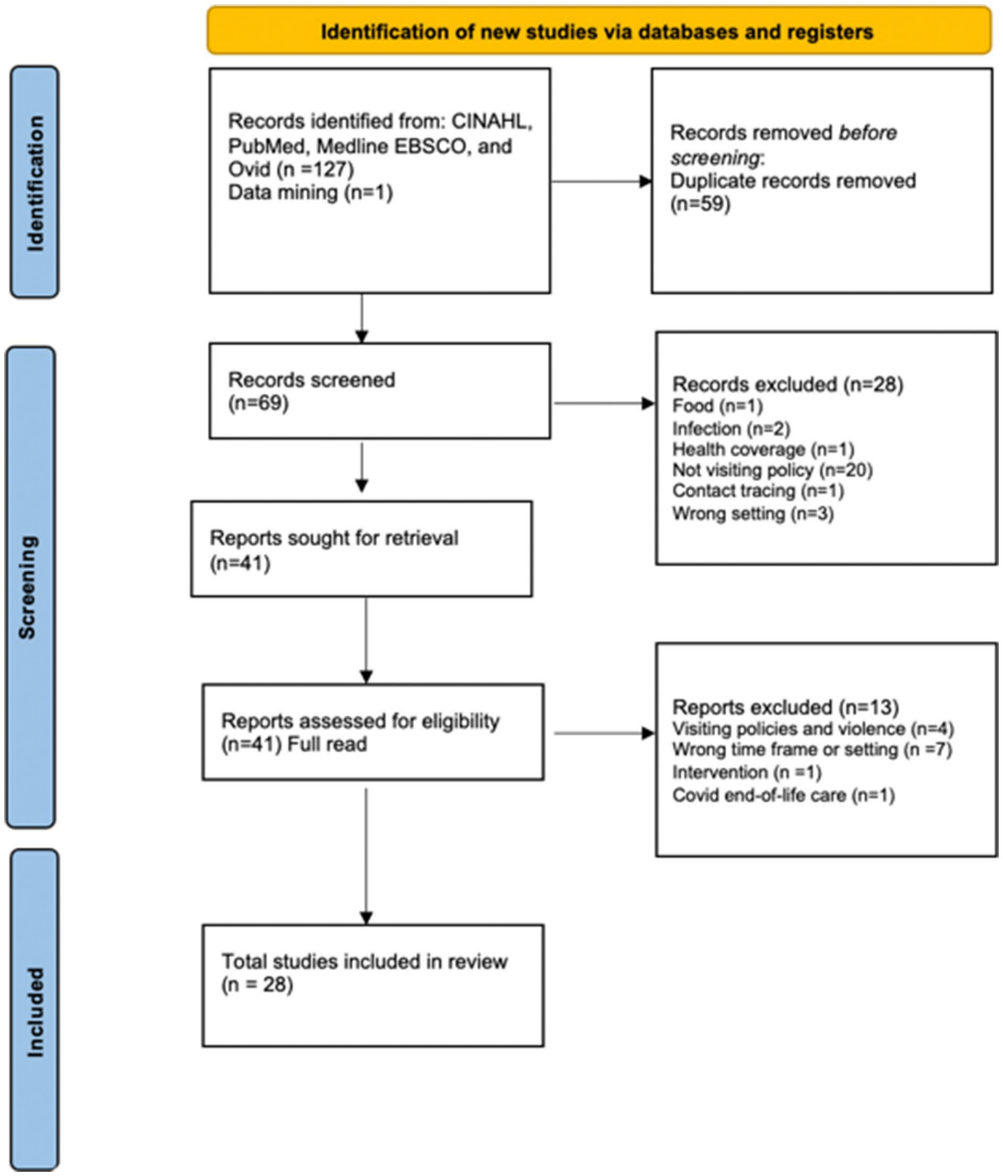
Integrative review of Covid-era visiting policy literature. *Consider, if feasible to do so, reporting the number of records identified from each database or register searched (rather than the total number across all databases/registers). **If automation tools were used, indicate how many records were excluded by a human and how many were excluded by automation tools. From Page et al. (18). *EBSCO = 47, Pubmed = 47, CINAHL= 30, Ovid= 4 **All exclusions made by human.

The search of CINAHL, PubMed, Medline EBSCO, and Ovid resulted in 127 studies being retrieved, with one article added through data mining. Fifty-nine duplicate articles were removed. A title search removed 28 articles, leaving 41 to read in full. Thirteen articles were then excluded after a full read. Four articles were excluded because they focused on visiting policies related to violence. Seven articles were excluded because they were in the wrong time frame (pre-Covid) or setting (long-term care or physician visits). One article was excluded because it was focused on family engagement as an intervention and did not include visiting policies; another was excluded because it was focused on the impact of Covid on end-of-life care and not on visiting policies. After reviewing the articles, the integrated review includes 28 articles. Figure 1 provides the PRISMA diagram of the screening of the documents for the integrated review (18).

## 5. Quality appraisal

An integrated review aims to provide the broadest research review (16). This review used the Johns Hopkins Nursing Evidence-Based Practice (JHEBP) tool (19). The JHEBP tool can be used to assess the hierarchy and quality of an article, with a level 1 given to randomized control trials and systematic reviews of randomized control trials, a level 2 designated for quasi-experiments, a level 3 for non-experimental study, a level 4 for opinions from respected authorities or committees, and finally, a level 5 for quality improvement projects, narratives, or case reports.

## 6. Data abstraction

Data were retrieved, assessed, and tabulated by one reviewer. The articles were evaluated for the study design, aim, conclusions, and insights into family engagement and patient-family-centered care. Additional insights and comments were included. The investigation details are captured in narrative form in results and matrix form in Tables 1, 2.

**Table 1:** Summary of included articles.

	Article (citation)	Study design/quality/ sample population	Aim	Conclusion	Family engagement domains	Other concepts
1	El Aziz et al. (20)	Retrospective comparative study to compare patient outcomes and length of stay before and after restrictive visiting policy Level 1c (insufficient sample size) United States 439 Patients from one hospital	Evaluate the impact of visitation policy (VP) on short-term surgical outcomes	Restrictive VP for colorectal surgery patients was not associated with in-creased postoperative complications and readmission rates. Length of stay (LOS) was similar between the two groups. More research needed to confirm findings.	Presence: suggestion to use novel techniques might optimize communication between patient and family (no mention of family-provider communication)	Low satisfaction scores a focus of the restrictive visiting policy Isolation
2	Ahmed et al. (21)	Descriptive, phenomenological qualitative study using interviews Level 3b UAE 37 ICU Nurse Managers from 13 non-government hospitals	Explore how nursing services were managed and provided in intensive care units during the COVID-19 pandemic Clarify the management lessons learned to inform future challenges to the visiting policy	Promising strategies for intensive care units in planning for responses to future crises include maximizing organization resources, boosting family-centered care, providing in-service training for nurses, and policy reform.	Communication: lack of family meetings had a negative influence on quality of care Use available technology to facilitate patient-family communication Shared decision-making/participation: lack of family involvement in decision-making process. Encourage patient virtual presence in rounds	
3	Akbari et al. (22)	Parallel randomized clinical trial Level 1b Iran 60 patients and 57 Nurses from 4 ICUs in 2 hospitals	Investigate relationship between increasing visitation time and patients’ physiological parameters in ICU Examine nurses’ beliefs and attitudes toward visiting	Increasing visitation times can lead to a positive effect on the patient’s physiological parameters. No significant change in nurse beliefs	Presence: nurses believed that family presence would cause physiological stress. It had a positive effect on patients’ physiological parameter	Visiting policy linked to outcomes and satisfaction
4	Azad (23)	Narrative of a neurocritical resident Level 5b United States 1 Physician from 1 hospital	Illustrate the disproportionate effect of restrictive visiting policies on traumatic brain-injured patients and their families. Catalyze discussions regarding the need for careful evaluation of restrictive family visitation policies and exceptions that may be required for patients with brain injuries	COVID-19-era family visiting policies negatively affect the healthcare team’s ability to partner with families in navigating complex decisions after brain injury.	Presence: families of neurocritical brain-injured patients are not permitted bedside after an initial 2-h visit allowance for one person. Communication/information sharing: difficult and complex conversation even more difficult without being bedside. It is difficult to convey heaviness of the situation on video Shared decision-making/participation: difficult for families to limit care when they do not get a complete sense of the injury Regular, in-person family meetings facilitate trust and information sharing and often cannot be digitally replicated	Exceptions to policy, Race and socioeconomic factors affect trust in healthcare system. Choice architecture: families felt the only way they would be allowed to visit is if they agreed to comfort measures only
5	Azad et al. (24)	Regression discontinuity and time-to-event analysis Level 2b United States 940 Patient (decedents) from 2 large academic hospitals	Determine if a restrictive visitor policy lengthened the decision-making process for dying inpatients	Policies restricting family presence may lead to longer ICU stays and delay decisions to limit treatment prior to death	Presence: visiting policy that restricts family presence has unintended consequences. Shared decision-making/participation: policy restricting family presence led to longer ICU stays and delayed decisions to limit treatment before death.	Exceptions to policy, visiting had a positive impact on shared decision making
6	Brauchle et al. (25)	Online survey of clinicians with a mixed methods approach Level 3b 5 German-speaking countries 385 multi-disciplinary Leading Clinicians (172 nurses, 213 physicians) from all 1,943 ICUs in Austria, Germany, Luxemburg, and parts of Switzerland	Provide insights into visiting policies and family-centered care practices with a focus on children as visitors in ICUs	Family-centered care policies had been implemented in their units, including open visiting policies, allowing children as visitors without age restriction, and other family-centered care activities.	Presence: only 1/3 of respondents reported that ICUs were open 24h, seven days a week. 65% of respondents report family is not present during invasive procedures Communication/information sharing: 50% report not adopting family participation in rounds, and 47% somewhat adopt structured family conferences. Shared decision-making/participation: 47% of respondents report only somewhat adopting goals of care meetings in the ICU Respect/family needs met: 41% of respondents report fully adopting open visiting hours that are flexible and culturally appropriate Contribution to care/participation: 40% of respondents reported somewhat adopting disseminating information and helping families in ways to assist the patient Collaboration: 70% report not adopting a family advisory group	Suffering when family, including children, cannot be there
7	Brown et al. (26)	Retrospective cross-sectional study Level 2b United States 2931 Patients from 4 hospitals	To explore if the restrictive visitation impacts use of benzodiazepines and antipsychotics in older adults in the hospital setting	Benzodiazepine use was lower in older patients on days when visitors were allowed	Presence: the absence of caregiver support may lead to increased delirium, agitation, or anxiety, leading to increased benzodiazepine use. Presence of a caregiver improves quality of medication prescribing Contribution to care/participation: caregivers improve orientation and reduce agitation	Isolation
8	Chan et al. (27)	Mixed methods study using online survey and open-ended questions describing how the services were affected Level 3b Hong Kong 142 multi-disciplinary Clinicians (Physician 24, Nurse 56) from public hospitals	Examine the mental health of palliative care professionals during COVID	82% felt stressed when communicating with patients and family members under the no-visiting policy during the pandemic Professionals identified tightening visiting policies as 1 of 3 themes that affect the provision of palliative care	Presence: the absence of family and caregivers led to increased loneliness and distress, which in turn affects the provider Respect/needs of the family met: difficult for provider to assess and provide support because of reduced interaction with family Contribution to care/participation: caregivers experience guilt because they are unable to fulfill caregiver duties	Isolation Loneliness Burnout (professional)
9	Chary et al. (28)	Commentary Level 5b United States 1 Physician from 1 hospital	To describe inconsistent visitor restriction policies	Inconsistent visitor policies contribute to health inequities among minority older adults with limited English proficiency	Presence: allowing exceptions for caregivers of patients with cognitive impairment. Health disparity for patients with limited English proficiency who did not know they could accompany the patient. Communication/information sharing: caregiver is seen as difficult when trying to advocate for patient when it may be related to mistrust. Caregivers offer important information for initial work-up. The process of conference calls with LEP family is time-consuming, inconvenient, and cumbersome Collaboration: visiting policies posted online in only English, even with the population most affected by Covid being Latinx.	Exceptions to policy are unfairly given and not in a systematic way; health equity cultural capital (social skills and connections that allow for advancement within institution), which includes health literacy, fluency in dominant language, confidence, and comfort in advocating for oneself; street level bureaucracy-front line staff as the gatekeeper
10	Chua et al. (29)	Multisite study identifying key elements of website manner that are helpful when conducting serious illness conversations by virtual visit Level 3c United States Unknown number of Clinicians in unknown number of hospitals	Explore the effectiveness of virtual palliative care	Like bedside manner, nuanced verbal and nonverbal web side manner skills are essential to conducting serious illness conversations during virtual visits.	Presence: virtual communication requires nuanced verbal and non-verbal skills like bedside manner. Communication/information sharing: thoughtful use of the virtual visit can pose a benefit for patients, families, and clinicians to facilitate serious illness conversations during social distancing and visitor restrictions Shared decision-making/participation: serious illness conversations require eye contact, lighting, a private setting, and intentionality to help establish rapport	Social, economic, technological, and demographic barriers
11	Creutzfeldt et al. (30)	Semi-structured interviews Level 3b United States 19 Family members from 1 hospital	Explore the experiences of family members of patients with severe acute brain injury focusing on the impact of family presence in the hospital.	Family presence at patient’s bedside fulfills important needs. Visitation restrictions require hospitals to be creative and inclusive to help maintain these connections.	Presence: being bedside helps the family Communication/information sharing: observing and listening to providers on rounds helps families cope, builds trust in the team, and shares clinical information. Regular standardized video communication can also accomplish this. Respect/family needs met: being bedside helps the family cope, advocate, and support patient. The family also receives support from other families and ICU staff.	Understanding the diversity of needs is an important step toward meeting those needs Distress
12	Danielis et al. (31)	Retrospective cohort study was conducted according to the “Strengthening the Reporting of Observational Studies” in Epidemiology statements Level 3b Italy 80 Patients from 1 postoperative surgical unit in 1 large academic center	Assess the consequences of hospital visitation restrictions on the most common outcome measures for adult patients who underwent surgery.	Visiting policy restrictions should be balanced between potential benefits (e.g., preventing negative outcomes on patients) and threats (e.g., increasing the spread of the virus).	Presence: patients that experienced no visitor policies were more likely to experience disorientation, restraint, and sleeplessness. Communication/information sharing: family members communicated patients’ pain to provider; therefore, the pain was better managed Contribution to care/participation: family, in addition to providing support and comfort to patients, might play an active role in early recognition of clinical deterioration and pain	
13	Dhawan et al. (32)	Cross-sectional analysis of visitation policies abstracted from public-facing Web sites of comprehensive cancer centers (CCCs) Level 5b United States 50 National Cancer Institute designated adult comprehensive cancer centers	Examine the availability of language translations of visitation restrictions on adult National Cancer Institute-designated CCCs websites.	Even in cities and states with larger Hispanic/Latinx populations, most CCCs did not publish resources in Spanish. This study highlights a key opportunity to mitigate communication barriers and deliver culturally competent, patient-centered care.	Presence: non-English-speaking families had to take additional steps to obtain the visiting policy. Respect/family needs being met: visiting restrictions impact communal medical decision-making for patients from allocentric cultures who value familism. Shared decision-making/participation: visiting policy is a barrier to high-quality communication needed in serious illnesses like cancer.	Cultural competence
14	Eden and Fowler (33)	Descriptive, exploratory survey design Level 3b United States 45 Family members from one hospital	Details a study of family members’ perceptions related to being isolated from hospitalized patients with confirmed positive COVID-19.	Most family members (89%) wanted to visit their loved one in the hospital, and the same amount called the patient or patient-care unit themselves.	Presence: emotional anguish when the family could not physically visit the patient. Communication/information sharing: family received limited information about their family member’s condition. One-quarter of family members recalled getting phone calls from staff with updates. No family member reported a “virtual visit” facilitated by the hospital staff	Isolation
15	Eugênio et al. (34)	Cross-sectional study Level 3b Brazil 95 Family members and 95 multi-disciplinary clinicians (19 nurses, 57 nursing technicians, and 11 physicians) from one clinical-surgical ICU in one hospital	Compares the perceptions of patients’ relatives with the perceptions of health professionals regarding a flexible visitation model in intensive care units.	Family members and staff-have different perceptions of flexible visits in the ICU. A positive view regarding the perception of decreased anxiety and stress among the family members and greater information and contributions to patient recovery predominates with a flexible visiting model.	Presence: having a companion bedside benefits the patient’s recovery and reduces anxiety and stress in patients and families Communication/information sharing: facilitates information exchange. Providers wanted education to help them communicate with families. Contribution to care/participation: presence of family allows them to be active agents in patient care and important ally in the team.	Burnout (professionals)
16	Fiest et al. (35)	Environmental Scan of Canadian hospital visitation policies during first wave of pandemic Level 5b Canada 257 Policy Documents of Canadian hospitals with adult ICUs	Describe the extent, variation, and fluctuation of Canadian adult intensive care unit (ICU) visitation policies before and during the first wave of the COVID-19 pandemic.	During the first wave of the COVID-19 pandemic, most Canadian hospitals had public-facing visitor restriction policies with specific exception categories, most commonly for patients at end-of-life.	Presence: alternative ways to connect (email, virtual, phone call) Communication/information sharing: dedicated team member to schedule and facilitate virtual visit Respect/family needs met: few policies allow for culturally appropriate practices or protocols. Also, no allowances for patients with prolonged stays.	Exceptions for end-of-life, critical illness, patients requiring assistance related to cognitive or physical disability
17	Fino et al. (36)	Cross-sectional study online survey of health- care workers probing on socio-demographic and work-related variables Level 3b Italy 209 multi-disciplinary Clinicians (146 Nurses, 63 physicians) from one region in country (“worst-hit”)	Investigate whether facilitating virtual patient-family communications would mitigate distress levels in engaged healthcare professionals.	Nurses assisting patient-family videocalls reported significantly lower levels of distress and a better quality of wakefulness than those who did not. In contrast, physicians reported higher levels of distress during such virtual communications.	Communication/information sharing: virtual communication technologies between families and providers need to be complemented with education for the providers on how to enhance, especially in terms of communicating online and on difficult topics	Isolation Burnout (professionals)
18	Kean and Milner (37)	Quality Improvement Project Level 5b United States 1 Adult ICU in one hospital	Develop and implement more family-centered visitation policies in the ICU	Evidence supports open visitation as best practice that aligns with patient/family-centered care and patients in adult ICUs. Resulting open visitation policy improved satisfaction among nurses, patients, and visitors.	Communication/information sharing: open visitation improves communication and understanding Respect/family needs met: patient has right to consent to visitor	There was conflict between nurses who made exceptions to the restricted visitor policy and those who did not.
19	Kim et al. (33)	Retrospective observational study using medical record review before covid (restrictive visiting policy) and (no-visiting policy) after covid Level 3b South Korea 2,196 Patients from one ICU in 1 hospital	Evaluate the effect of intensive care unit (ICU) visit on the incidence of delirium, delirium subtype, and anxiety level in ICU patients.	The no-visiting policy during pandemic did not affect the incidence of delirium. The proportion of patients with hyperactive or mixed delirium was higher during the no-visiting period. No visiting was a risk factor for the non-hypoactive delirium subtype and high anxiety levels.	Presence: there was no difference in the incidence of delirium, regardless of whether the visit was allowed. A potential explanation is that the limited daily visiting hours of restrictive ICU visits might be too short to help reduce the incidence of delirium. This finding confirmed the importance of family visits in changing delirium subtypes and alleviating anxiety in ICU patients and provided a foundation for nonpharmacological intervention in the ICU.	Isolation
20	Maloh et al. (38)	Cross-sectional, descriptive, and comparative survey design Level 3b Jordan 234 Nurses from 5 hospitals (1 public and 4 private)	Evaluate nurse managers’ and nurses’ perspectives on the effects of an open visitation policy at intensive care units (ICUs) on patients, families, and nurses’ care.	ICU managers and staff nurses do not favor implementing an open visitation in their units despite its known benefits, international recommendations, and relevance and compatibility with the local religious and cultural context.	Presence: managers believed that family interfered with nursing care. Communication/information sharing: the second most agreed-upon belief was that a more flexible policy would cause nurses to spend more time providing information to the family and interfere with communication between nurses. Respect/family needs met: most nurses also believed that the visiting policy did not need to be adapted to be more culturally appropriate. In the Arabic and Muslim environment, social bonds and family connection are important; family members, friends, and coworkers are expected to assist critically ill family members.	
21	Marmo and Milner (39)	Mixed-methods study Level 3b United States 96 Hospitals (Magnet and Pathway to Excellence)	Compare visitation policies of Magnet and Pathway to Excellence hospitals with pre-pandemic open visitation in adult intensive care units.	Despite evidence supporting the benefits of visitation and the harms of restricted visitation and expert recommendations for returning safe visitation to hospitals, Magnet and Pathway to Excellence hospitals continue to enforce restricted visitation policies in intensive care units.	Presence: a common theme was that visitors were not welcome even though the nurses reflected that not having visitors does cause harm to the patient, family, and staff, which they separated from the harm of the virus. Communication/information sharing: changes in visitation policy, with subthemes of technological and nontechnological adaptations to nursing work to facilitate communication with family. Contribution to care/participation: no overnight visitation was allowed unless the visitor was essential to the patient’s care.	Exceptions for patients at the end of their life. A culture shift from open to restricted visitation and the use of evidence-based practices to improve patient care outcomes.
22	Padua et al. (40)	Quasi-experimental study Level 2b Italy 11 Patients from one ICU in 1 hospital	Assess whether digital communication benefits patients with disorders of consciousness (DOC), considering the sensory and emotional deprivation due to the COVID-19 emergency lockdown.	“Digital re-connection” is needed, especially for fragile population groups such as patients with DOC.	Presence: patients experienced an autonomic activation with both visual-audio interactions with family.	
23	Rosa et al. (41)	Cluster-randomized crossover trial as a secondary analysis of the ICU Visits Study Level 1b Brazil 863 Family members from ICUs in public and private nonprofit hospitals with 6 or more beds	Investigate whether the effect of a flexible ICU visiting policy that includes flexible visitation plus visitor education on anxiety symptoms of family members is mediated by satisfaction and involvement in patient care.	Flexible ICU visiting policy reduces anxiety symptoms among family members and increases satisfaction. Increased participation in some patient care activities because of flexible visitation was associated with higher severity of anxiety symptoms.	Presence: flexible visiting policies and proximity improves anxiety symptoms by increasing satisfaction Communication/information sharing: by meeting common family needs during ICU stay, such as proximity with the patient, better communication, and reassurance. Flexible visiting policy might reduce uncertainty about patient survival, effective management, comfort, and risk of significant disability. Contribution to care/participation: higher involvement in care in ICU, associated with flexible visitation, is also associated with higher severity of anxiety symptoms in families.	Burnout (professional)
24	Segar (42)	Narrative Level 5b United States 1 Physician from 1 hospital	Share narrative of physician experience with visiting policy and a dying patient	A narrow view of patients’ and families’ preferences has led to unjustly applying policies to accommodate dying patients.	Presence: family bedside is beneficial to patients, families, and providers. Providers experience distress when they know it benefits the patient and family but are forced to turn the family away. Presence is therapeutic for the management and prevention of delirium in the patient. Communication (information sharing): poor quality of communication when technology is the only interaction with the family. Giving the family bad news over the phone is additionally distressing for providers. Shared decision-making/participation: restrictive visiting policy delays family conferences Respect/family needs being met provider worries about providing a culturally appropriate death and respects the wishes of the family and patient within the confines of the policy.	Exceptions create choice architecture and are unfairly given Isolation Distress
25	Shinohara et al. (43)	Comparative study Level 2b Japan 200 Patients from 1 ICU in 1 hospital	Investigate the association between the no-visitation policy and delirium in intensive care unit (ICU) patients	No-visitation policy was not associated with the development of delirium in ICU patients.	Presence: no relationship between the number of days until the development of delirium and the no visitation policy in this study. The patients may not recognize the visitors because of poor consciousness which may reduce the effectiveness of visits to prevent delirium.	
26	Suh et al. (44)	Cross-sectional descriptive survey Level 3b South Korea 99 Family members from 1 adult ICU from 1 academic medical center	Compare the quality of life, depressive symptoms, and emotions in family caregivers of ICU patients before and during the COVID-19 pandemic and explore families’ perceptions and suggestions for the visitation.	Visitation restriction is necessary during the COVID-19 pandemic despite sadness and anxiety reported by caregivers. Hence, alternative visitation strategies are needed to mitigate psychological distress and provide sufficient information to ICU family caregivers.	Presence: families reported being sadder and more anxious than before the restrictive vising policies. They also felt that the separation had adverse consequences. Communication/information sharing: only half of families felt they were kept informed of their family member’s condition. Respect/family needs met: family reported not having enough information about their family member’s medical condition and treatment plan. Collaboration: the respondents suggested more frequent meetings with clinicians, offered alternative contact methods with the patients, and improved orientation of the family visitation policy.	
27	Wasilewski et al. (45)	Qualitative descriptive study Level 3b Canada 10 Patients, 5 family members, and 12 Clinicians (2 nurses) from 1 hospital network system	Explore how infection control measures impacted stakeholders’ perceptions of care quality and interactions with others and investigate how these experiences and perceptions varied across stakeholder groups.	Infection control challenged psychosocial health and maintenance of vital human connections. All stakeholders experienced loneliness and isolation as well as COVID-related stigma. Technological innovations mitigated some of the isolation. The study underscores the need to balance safety with well-being of all stakeholders.	Presence: participants spoke of the pronounced isolation, loneliness, and need for human connection. Absence of family meant that patients did not have someone readily available for emotional support. Respect/family needs met: not all families have equitable access to the technology that helps connect families and patients. Infection control and prevention measures perpetuated the COVID-related stigma that stakeholders experienced.	Participants in our study were English-speaking and had mid-to-high socioeconomic status (SES). Isolation Loneliness
28	Zeh et al. (46)	Pre- and post-retrospective cohort novel survey study Level 3b United States 117 Patients from 1 academic center	Understand the impact of visitor restriction rules on the postoperative experience of patients undergoing surgery.	Implementation of restrictive visitor policies may adversely impact the post-operative experience of Covid-negative patients undergoing surgery and highlight the need for patient-centered strategies to improve the postoperative experience of patients during ongoing or future disruptions to routine hospital practices	Presence: patients indicated that they rely upon family for social support and that without them, they were lonely and felt isolated Contribution to care/participation: some patients in the No-Visitor Cohort felt that their visitors provided direct support. They were less likely to report timely access to pain, nausea, and other medications and help to get out of bed. Respect/family needs met: patients in the No-Visitor Cohort were less likely to strongly agree that their and family members’ preferences were adequately considered upon discharge.	Decrease in hospital satisfaction at least partially related to the absence of visitors Isolation Loneliness

**Table 2:** Summary grid of sample and family engagement domains present in the literature.

		Country	Level of Evidence Using JHEBP	Population and sample size	Presence	Com-muni-cation	Shared Decision-making	Contribute to care	Family needs	Col-labor-ation	Link to sat-faction	Link to out-comes
1	El Aziz et al. (20)	United States	1c	439 Patients	X						X	X
2	Ahmed et al. (21)	UAE	3b	37 ICU Nurse Managers		X	X					
3	Akbari et al. (22)	Iran	1b	60 Patients 57 Nurses	X						X	X
4	Azad (23)	United States	5b	1 Physician	X	X	X					
5	Azad et al. (24)	United States	2b	940 Patients	X		X					X
6	Brauchle et al. (25)	5 German-speaking countries	3b	385 Clinicians	X	X	X	X	X	X		
7	Brown et al. (26)	United States	2b	2,931 Patients	X		X	X				X
8	Chan et al. (27)	Hong Kong	3b	142 Clinicians	X		X	X	X			X
9	Chary et al. (28)	United States	5b	1 Physician	X	X				X		
10	Chua et al. (29)	United States	3c	Clinicians unknown	X	X	X					
11	Creutzfeldt et al. (30)	United States	3b	19 Family members	X	X			X			
12	Danielis et al. (31)	Italy	3b	80 Patients	X	X		X				X
13	Dhawan et al. (32)	United States	5b	50 Comprehensive cancer centers	X		X		X			
14	Eden and Fowler (33)	United States	3b	45 Family members	X	X						
15	Eugênio et al. (34)	Brazil	3b	95 Family members 95 Clinicians	X	X			X			X
16	Fiest et al. (35)	Canada	5b	257 Policy Documents	X	X			X			
17	Fino et al. (36)	Italy	3b	209 Clinicians		X						X
18	Kean and Milner (37)	United States	5b	1 ICU		X			X			
19	Kim et al. (33)	South Korea	3b	2,196 Patients	X							X
20	Maloh et al. (38)	Jordan	3b	234 Nurses	X	X			X			
21	Marmo and Milner (39)	United States	3b	96 Hospital	X	X		X				
22	Padua et al. (40)	Italy	2b	11 Patients	X							X
23	Rosa et al. (41)	Brazil	1b	863 Family members	X	X		X			X	X
24	Segar (42)	United States	5b	1 Physician	X	X	X		X			X
25	Shinohara et al. (43)	Japan	2b	200 Patients	X							X
26	Suh et al. (44)	South Korea	3b	99 Family members	X	X			X	X	X	X
27	Wasilewski et al. (45)	Canada	3b	10 Patients 5 Family member 12 Clinicians	X				X			
28	Zeh et al. (46)	United States	3b	117 Patients TOTAL 6,984 Patients 1,126 Family members 1,174 Clinicians (780 nurses, 314 physicians) 96 Hospitals 50 Cancer Centers 1 ICU 257 Documents	X			X	X		X	X

This data abstraction and evaluation was approached using the Whittemore and Knafl methodology (16). This review of quantitative, qualitative, mixed methods research and narratives were assessed considering the pandemic-era hospital visiting policy on family engagement and patient-family-centered care for an older and seriously ill patient. Taking guidance from Whittemore and Knafl (16), the researcher extracted data, noting patterns and themes, common and unusual patterns, to build a description of family engagement in the hospital. Any questions that arose in the data abstraction process were discussed with all authors to reach a consensual decision.

## 7. Results

### 7.1. Diverse perspectives, designs, and sample population

The integrative review represents an international perspective, as captured in Table 2. Almost half of the twenty-eight studies illustrate the visiting policies in the United States. There are two Canadian studies and two Brazilian studies. There are three Italian studies and one study representing five countries with individuals who are literate in and speak German. There are two South Korean, one Japanese, and one Chinese territory, Hong Kong studies. Finally, there are studies from the United Arab Emirates, Iran, and Jordan that represent a Middle Eastern perspective. There are no African countries or Australian studies described in the literature.

The review includes diverse study designs, including quantitative, qualitative, and mixed-method designs. Seven studies include a survey in their design (12, 25, 27, 33, 36, 38, 46). The review includes randomized control trials (22, 41), retrospective comparative studies (20, 26, 31, 38, 43, 47), observational studies (29, 40), and a regression discontinuity and time to event design (24). There are qualitative studies with semi-structured interviews (21, 30, 45). There are two mixed-methods studies (27, 39), three narratives (23, 28, 42), an environmental scan (35) and an analysis on primary language availability of patient-facing policy (32).

The articles were critically appraised using the Johns Hopkins Nursing Evidence-Based Practice (JHEBP) tool (19). The articles range from a rating of 1b to a 5b, with most articles falling in the 3b range. Two randomized control trials are rated 1b (22, 41), and a retrospective comparative study is rated 1c (20). Six articles are rated 5b (23, 28, 32, 35, 37, 42).

As well as having an international perspective and a diverse collection of study designs, this review provides a multidisciplinary perspective. The review presents the nursing, physician, health policy, and social work perspectives, to name a few. One-third of the articles are published in nursing journals. Another third of the articles are published in multidisciplinary journals. The remaining third are published in medical journals.

The review encompasses a large and diverse sample population. The perspective most captured is that of the patient. There are close to 7,000 patients included in the review. The second most substantial perspective is that of the provider. There are 1,174 multidisciplinary clinicians included in the review. Of the over one thousand clinicians, 780 are nurses, and 314 are physicians. One study does not share the number of clinicians (29). The review includes 96 hospitals, 50 comprehensive cancer centers, and one hospital unit. In addition, the review includes 257 policy documents.

### 7.2. Family engagement domains

**Figure 2:**
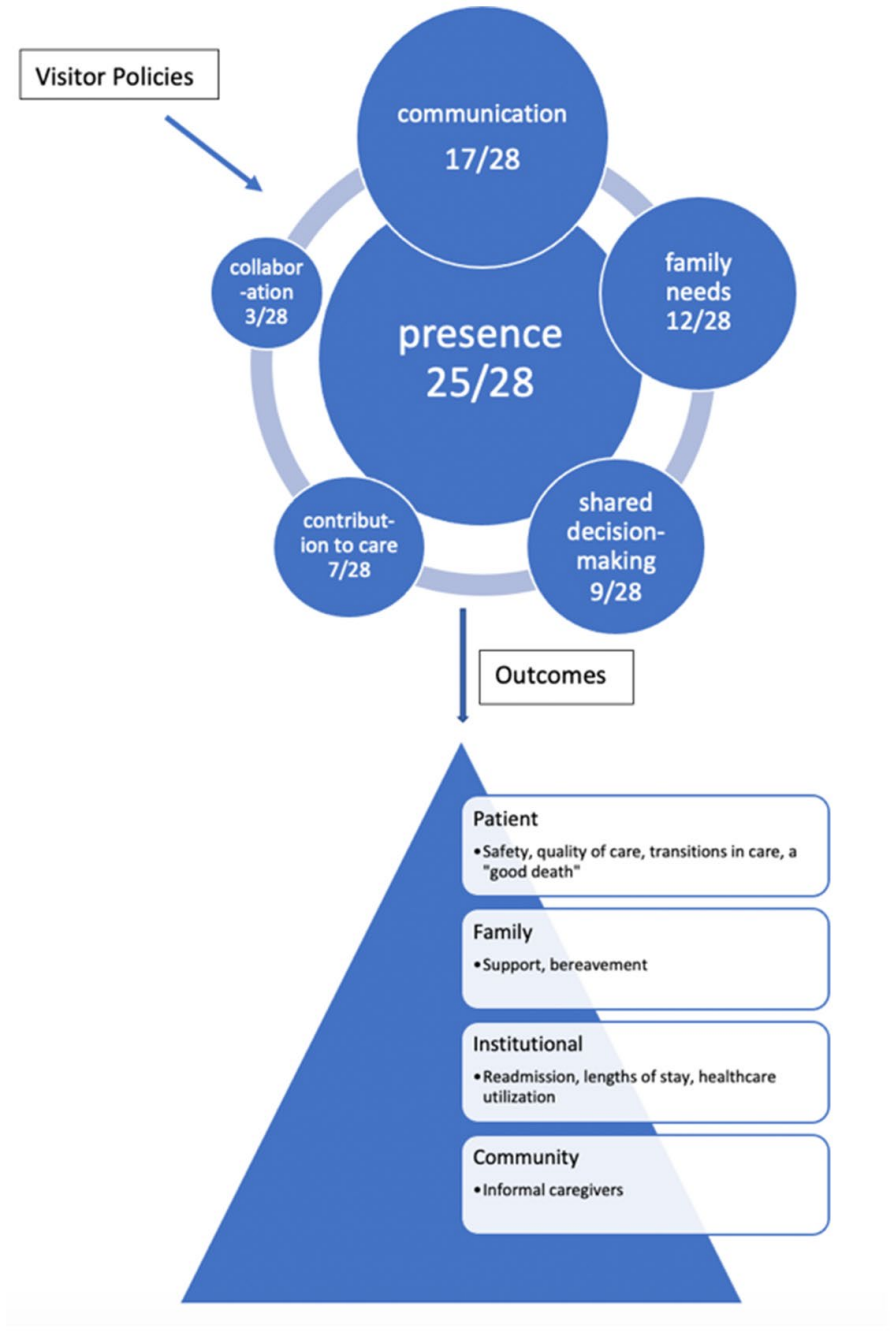
The frequency and variability of the domains of family engagement within the visiting policy literature and the outcomes that are affected.

Assessing the articles on visiting policies for evidence of the domains of family engagement and patient-family-centered care reveals large variability between domains that the studies convey and those domains that studies do not. The most noticeable domains in the studies are presence, communication (information sharing), and shared decision-making (participation). Figure 2 illustrates the variability of the domains. Twenty-five of the twenty-eight, or 90%, articles on visiting policies connect visiting to the family engagement domain of presence. The family engagement domain of communication overlaps with the patient-family-centered care domain of information sharing. The next most prominent domain linked to visitor policies in 60% of the articles included in the review is communication or information sharing. Family engagement’s domain of family needs, like patient-family-centered care’s respect, is illustrated in a little less than half of the included articles.

Family engagement’s shared decision-making, contribution to care, and collaboration are the more active and the least present domains in the review. The interplay of shared decision-making, participation, and restrictive visiting policies are characterized in nine studies, or 32% of the included articles. The most active domain of family engagement, contribution to care, which shares some of the same principles of family patient-centered care’s participation, is described in only a quarter of the articles. Patient-family-centered care’s collaboration of family at the institutional level in policies and programs is the most absent domain and only portrayed in three articles, or 10% of the articles.

The articles in the review of visitors’ policies, family engagement, and patient-family-centered care primarily focus on patient outcomes. A little more than half of the articles link the policy and domains to outcomes (12, 20, 22, 24, 26, 27, 31, 34, 36, 40–43, 46, 47). These articles add to the body of evidence that supports the benefits of family engagement. Five articles connect the policy and domains to patient and family satisfaction (12, 20, 22, 41, 46).

### 7.3. Question of equity of visiting policy

Some of the twenty-eight articles identify other confounding factors to visiting policy and family involvement. The idea of exceptions to the visiting policy was raised in seven articles (22, 23, 28, 35, 37, 39, 42). Choice architecture, the context in which family and patient decisions are influenced (Blumenthal-Barby and Opel, 2018) is described in two articles (23, 42). The review also identifies the need to focus on health equity in eight articles (23, 28–30, 32, 35, 42, 45).

## 8. Discussion

### 8.1. Insights gained from integrative review

The literature on post-Covid era visiting policy adds to what is known about the benefits of family engagement for the patient by focusing on physical and emotional outcomes. For example, family presence improves the patient experience and outcomes, decreases loneliness, anxiety, and distress and mitigates certain types of delirium, and improves patients’ orientation and decreases their agitation (26). 8/16/23 4:09:00 PM While they provide emotional support and comfort to the patient, families also are active agents in the early recognition of clinical deterioration and pain (31, 46). Presence also positively impacts family members (30, 33, 34, 42, 44). Finally, several studies encourage leveraging technology to create a family-patient connection with phone, email, or video contact when physical presence is impossible (20, 21, 35).

The literature reviewed herin also adds to the interconnectedness of the domains and the impact that restrictive policies may have on equitable and culturally appropriate competent care. It is difficult for providers to assess and meet the family’s needs when they have reduced interaction with their families (27). Furthermore, few restrictive visiting policies are culturally sensitive (25) and may not be equitable. For one, not all families have the same access to technology and, therefore, cannot engage in the technology that can facilitate presence (45). Restrictive visiting policies impact culturally appropriate decision-making for patients who are members of cultures that value communal decision-making and families (32). Additionally, families report not having enough information about their loved one’s condition and treatment plan (44) or having their preferences considered for discharge planning (46). At the end of a patient’s life, clinicians worry that they cannot provide a patient with a culturally appropriate death that respects the patient’s and family’s wishes (42).

The literature recognizes that communication between providers and families can be complex, and the post-Covid era visiting policies increase the complexity. In the best of circumstances, presence sets the stage for information sharing between the family and the providers facilitating communication and partnerships between the family and the providers. Clinicians share information, educating patients and families on conditions, treatments, and care after discharge. Family members provide valuable contextual information to providers (28). Additionally, when families can ask providers questions or observe rounding, their understanding and knowledge about the patient’s condition increases (33, 37, 44). Communication builds a family’s trust in the provider (23, 28, 30). Post-Covid technological adaptations to enable communication with families have altered the technological demands of nursing work (39). However, adopting new practices that encourage family participation, like facilitating and scheduling conference calls with families of patients, especially for families with language barriers, can be time-consuming for the nurse and other providers (28) and therefore are frequently not incorporated into the work-flow (25).

When families cannot be present because of restrictions or physical distance, the literature shares that technology may offer a solution, albeit not a perfect one. In the hospital, communication is fundamental to shared decision-making (7) and participation (9). Virtual family presence on rounds is encouraged when physical presence is impossible (21). The inability to have family bedside delays shared decision-making and may unnecessarily extend a patient’s stay in the intensive care unit (24, 42). However, the research is showing that providers need even more advanced communications education and training to establish a rapport and facilitate difficult conversations on a video platform (29, 34, 36) technology cannot wholly replicate in-person interaction, especially for serious illness and complex conversations (23).

Contribution to care and collaboration are poorly represented in the literature examining family engagement domains and visiting policy. These domains reflect the family as an active stakeholder in the patient’s care. Most clinicians report that their institutions do not have a family advisory group to help inform policies (26). Some of the effects of not having active partnerships with family can be seen in the literature. For example, even when the population most affected by Covid was the Latinx population; the online patient-facing visiting policy was only posted in English (28). In the clinical setting, patients and their families recommended that with the restricted visiting policies in place, providers should schedule more frequent family meetings with clinicians (44).

The literature also shows that family engagement is poorly operationalized. While literature adds more evidence to the benefits of family engagement, there are gaps in the description of family engagement in the hospital, how to operationalize it, and measure it. For example, while alternative methods of communication like phone or video can be used to update families on the patient’s condition and shared decision-making, few families report receiving telephone or video calls (33) even though the research encourages having a dedicated team member schedule and facilitate these conversations (35).

One of the consequences of not operationalizing family engagement is making exceptions to the visiting policy without systemization (28). Frontline workers are charged with enforcing the policy or making exceptions. Exceptions are made for patients at the end of their life, critical illness, or patients that require assistance for a cognitive or physical disability (35, 39). Unfortunately, the exceptions create choice architecture and are unfairly given (42). Exceptions affect the family’s trust in providers and the healthcare system (23). The exceptions also create conflict between the nurses who make exceptions and those who do not (37). Further evidence of the need to operationalize family engagement is the absence of a consistent proxy measure.

## 9. Limitations and strengths

There are limitations to this integrative review. First, the review covers a concise time frame from 2020 to 2023. Also, the visiting policies have undergone iterative changes within this time related to the uptake of vaccines and as more is learned about the virus. Additionally, the search process may not have captured everything to know. The Family Engagement Construct, while defined, includes many of the same concepts as the Patient-Family-Centered Construct, which may lead to some confusion and is behind the reason for combining the two. A final limitation is that one reviewer completed the review and assessment of the literature.

The review’s strengths lie in what we learn about family engagement in the hospital. The manuscript synthesizes the literature to reveal the foundational pieces that are most visible and strong in the hospital. It also identifies which domains could be strengthened. This review also reflects an international and multidisciplinary perspective of the care of seriously ill patients. Finally, this review is the first step toward operationalizing family engagement in the hospital setting, which could benefit all stakeholders.

## 10. Conclusion

This integrated review can guide practice, educational initiatives, and future research, for students, providers, and policy creators, by identifying the domains of family engagement and patient family-centered care related to the visiting policy. In practice, awareness of family engagement as a vehicle for improving outcomes is the first step to activating it. Presence which has been identified as one of the family engagement domains, may be the domain upon which all the others are built. It facilitates communication, family needs assessment, shared decision-making, and contribution to care. When presence is challenged by visiting policies or distance from family, nursing and other providers can leverage technology to mediate presence when it is impossible. Those who provide care can use the results of the review to activate family engagement domains that need support. Increasing awareness of the benefits of engagement helps create an institutional culture that recognizes the importance of encouraging and facilitating family engagement.

This review reveals opportunities for healthcare models and systems. The domain that offers the most significant opportunity for growth is collaborating with the stakeholders in the institutional policies. Collaboration is necessary to form partnerships between providers and families supporting the patient, family, community, and hospital. In the post-pandemic era, with capacity and workforce challenges, partnerships that reduce readmissions and healthcare utilizations become even more essential. Hospital policy creators can use the results of the review to guide policy creation that supports all the domains of family engagement.

Finally, further research on family engagement in the hospital setting is needed to operationalize family engagement. Describing the extent to which all the domains are seen within the hospital setting and identifying proxies to measure the domains would help test the effectiveness of family engagement interventions. Also, more information is needed to explore if institutional policies, like visiting policies, disproportionately burden specific populations, thereby increasing health disparities. Finally, research is required to explore the role that unmet social determinants of health may play in the ability of families to engage with a hospital patient.

## Author contributions

JM conceptualized, performed data abstraction and analysis, and wrote the manuscript. PG guided the review process, edited the manuscript, and provided guidance on figures and tables. LH helped guide the review process and gave manuscript edits. All authors contributed to the article and approved the submitted version.

## Conflict of interest

The authors declare that the research was conducted in the absence of any commercial or financial relationships that could be construed as a potential conflict of interest.

## Publisher’s note

All claims expressed in this article are solely those of the authors and do not necessarily represent those of their affiliated organizations, or those of the publisher, the editors and the reviewers. Any product that may be evaluated in this article, or claim that may be made by its manufacturer, is not guaranteed or endorsed by the publisher.
